# Monitoring Training Load to Understand Fatigue in Athletes

**DOI:** 10.1007/s40279-014-0253-z

**Published:** 2014-09-09

**Authors:** Shona L. Halson

**Affiliations:** AIS Physiology, Australian Institute of Sport, PO Box 176, Belconnen, ACT 2616 Australia

## Abstract

Many athletes, coaches, and support staff are taking an increasingly scientific approach to both designing and monitoring training programs. Appropriate load monitoring can aid in determining whether an athlete is adapting to a training program and in minimizing the risk of developing non-functional overreaching, illness, and/or injury. In order to gain an understanding of the training load and its effect on the athlete, a number of potential markers are available for use. However, very few of these markers have strong scientific evidence supporting their use, and there is yet to be a single, definitive marker described in the literature. Research has investigated a number of external load quantifying and monitoring tools, such as power output measuring devices, time-motion analysis, as well as internal load unit measures, including perception of effort, heart rate, blood lactate, and training impulse. Dissociation between external and internal load units may reveal the state of fatigue of an athlete. Other monitoring tools used by high-performance programs include heart rate recovery, neuromuscular function, biochemical/hormonal/immunological assessments, questionnaires and diaries, psychomotor speed, and sleep quality and quantity. The monitoring approach taken with athletes may depend on whether the athlete is engaging in individual or team sport activity; however, the importance of individualization of load monitoring cannot be over emphasized. Detecting meaningful changes with scientific and statistical approaches can provide confidence and certainty when implementing change. Appropriate monitoring of training load can provide important information to athletes and coaches; however, monitoring systems should be intuitive, provide efficient data analysis and interpretation, and enable efficient reporting of simple, yet scientifically valid, feedback.

## Background

As athletes strive to improve their performance, modifications in training load are required, particularly increases in frequency, duration, and intensity. Training loads are adjusted at various times during the training cycle to either increase or decrease fatigue depending on the phase of training (i.e. baseline or competition phase). Ensuring that fatigue is titrated appropriately is important for both adaptations to training as well as for competition performance [1].

Fatigue is a complex and multifaceted phenomenon that has a variety of possible mechanisms. Indeed, a number of different definitions of fatigue exist, often dependent upon the experimental model employed and/or the conditions under which they occur. One of the most common definitions of fatigue was proposed by Edwards [2], and states that fatigue is a “failure to maintain the required or expected force (or power output).” Fatigue can also be influenced by the type of stimulus (voluntary or electrical), type of contraction (isometric, isotonic, and intermittent or continual), duration, frequency and intensity of exercise, and type of muscle [3]. Further, the physiological and training status of the athlete and the environmental conditions may also significantly influence fatigue. The definitions and caveats mentioned above highlight both the multi-factorial nature of fatigue and the inherent complexities of trying to monitor or measure fatigue in the athlete. For the purpose of this review, and to reflect a practical perspective, fatigue will be defined as “an inability to complete a task that was once achievable within a recent time frame” [1].

Monitoring the training load of an athlete is viewed by many as important to determine whether an athlete is adapting to the training program and to minimize the risk of non-functional overreaching (fatigue lasting weeks to months), injury, and illness. To date, research in this area is limited and much of what we know about monitoring comes from personal experience and anecdotal information. While monitoring within elite and professional sport is often extensive, much of these data remain protected and unpublished.

The objective of this article is to describe the current scientific information available relating to tools for monitoring training load in athletes and to outline some of the practical considerations when both planning and implementing monitoring in athletes.

## Reasons For and Against Monitoring Training Load

As mentioned above, there are a number of reasons why monitoring training load has become a modern, scientific approach to understanding athletes training responses and competition readiness. Monitoring training load can provide a scientific explanation for changes in performance. This can aid in enhancing the clarity and confidence regarding possible reasons for changes in performance and minimizing the degree of uncertainty associated with the changes. From these data, it is not only possible to retrospectively examine load–performance relationships, but also to enable appropriate planning for training loads and competitions. Importantly, load monitoring is also implemented to try to reduce the risk of injury, illness, and non-functional overreaching. Data may also be useful for team selection and determining which athletes are ready for the demands of competition.

There are also a number of benefits related to communication and relationship building with athletes, support staff, and coaches. When athletes are involved in monitoring, this can enhance their feeling of involvement in the training program, empower them, and increase their sense of ownership. Data collected from training monitoring can also be useful to facilitate communication between the support staff and coaching staff. When combined, these benefits can help enhance the belief and confidence associated with the training program.

However, not all coaches and scientists engage in athlete monitoring. For some athletes/teams/squads, insufficient resources can be a major reason for not including a system of training monitoring. Resources may be in the form of time, money, or the human resources needed to collect, process, and analyze the data. Further, there are no guarantees that monitoring training load will result in successful performances, therefore the resources required may not be provided. A lack of knowledge or experience with monitoring techniques can result in an inability to implement a practical and sustainable system and/or an inability to interpret the data collected. In addition, a clear rationale identifying *why* the monitoring is occurring, *what* will be monitored, how *often* monitoring will occur, and *how* the data are interpreted and presented back to the coaching staff is required. Finally, the ability and opportunity to implement change and provide feedback is critical to a successful monitoring system, and, if this does not occur, many attempts at monitoring are not sustainable [1].

## Potential Load Monitoring Measures

In order to gain an understanding of the training load and its effect on the athlete, a number of potential markers are available to athletes, coaches, and scientists. However, very few of these markers have strong scientific evidence supporting their use, and there is yet to be a single, definitive marker of fatigue described in the literature. Given the definition described in Sect. 1, it would appear that the best test of fatigue in terms of ecological validity would be a maximal performance test replicating the athlete’s event/competition. However, there are numerous difficulties regarding maximal testing in athletes. Maximal tests may add to existing fatigue in an athlete, which may be problematic around competition phases [4]. A taper may also be required to determine true performance capabilities, which is often impractical. When fatigued, athletes may also lack motivation to produce a maximal effort that is not for competitive purposes. For many sports, particularly team sports, it is extremely difficult to replicate or even define maximal performance [5]. Finally, if only maximal performance is assessed, little information can be gained regarding the potential mechanism/s of fatigue.

Table 1 outlines a number of variables that can be used to monitor training load and the resultant fatigue.

**Table 1:** Variables that can be used to monitor training load and subsequent fatigue

Variable	Units/descriptors
Frequency	Sessions per day, week, month
Time	Seconds, minutes, hours
Intensity	Absolute, relative
Type	Modality, environment
Maximal effort	Maximum mean power, jump height
Repeat efforts	Number of efforts, quality of efforts
Training volume	Time, intensity
Perception of effort	RPE
Perception of fatigue and recovery	Questionnaires; REST-Q, VAS
Illness	Incidence, duration
Injury	Type, duration
Biochemistry and hormone analysis	Baseline, response to exercise
Technique	Movement deviations
Body composition	Total body weight, fat mass, fat-free mass
Sleep	Quality, quantity, routine
Psychology	Stress, anxiety, motivation
Sensations	Hopeful, neutral, hopeless

*REST-Q* Recovery Stress Questionnaire, *RPE* rating of perceived exertion, *VAS* visual analog scale

## Internal versus External Load

When monitoring training load, the load units can be thought of as either external or internal. Traditionally, external load has been the foundation of most monitoring systems. External load is defined as the work completed by the athlete, measured independently of his or her internal characteristics [6]. An example of external load in road cycling would be the mean power output sustained for a given duration of time (i.e. 400 W for 30 min). While external load is important in understanding work completed and capabilities and capacities of the athlete, the internal load, or the relative physiological and psychological stress imposed is also critical in determining the training load and subsequent adaptation. As both external and internal loads have merit for understanding the athlete’s training load, a combination of both may be important for training monitoring. Indeed, it may be the relationship between external and internal loads that may aid in revealing fatigue. For example, using the cycling external load mentioned above, the power output may be maintained for the same duration; however, depending on the fatigue state of the athlete, this may be achieved with a high or low heart rate or a high or low perception of effort. It is this uncoupling or divergence of external and internal loads that may aid in differentiating between a fresh and a fatigued athlete [1].

## Methods for Monitoring External Load

### Power Output, Speed, and Acceleration

To gain an understanding of external training load, a number of technologies are available to athletes and coaches. In the sport of cycling, power output-measuring devices such as SRM™ and PowerTap™ allow the continuous measurement of work rate (power output) [7]. Training and competition can be recorded and data can be analyzed to provide information on a number of parameters, including average power, normalized power, speed, and accelerations. Cycling power output can be converted into a Training Stress Score™ (TSS™) via commercially available software [1] and allows the quantification of training based on relative intensity, duration, and frequency.

### Time–Motion Analysis

In team sports, time–motion analysis (TMA), including global positioning system (GPS) tracking and movement pattern analysis via digital video (such as ProZone™) are becoming increasingly popular to monitor athletes [5], particularly during competition. The reliability of GPS for monitoring movement is influenced by factors such as sample rate, velocity, and duration and type of task [8]. From the available literature, it appears that the higher the velocity of movement, the lower the GPS reliability [8]. Further, the reliability is also reduced when assessing tasks that require a change of direction and GPS does not quantify the load of jumping, kicking the ball, and tackling actions [8]. Typically, when using TMA for monitoring, arbitrary speed thresholds are set [9]. These categories may include walking, jogging, running, striding, sprinting, etc. [8]. It is becoming increasingly popular to associate TMA data with arbitrary and individualized speed thresholds. Lovell and Abt [9] compared TMA data from video analysis as arbitrary units with units expressed as individual speed thresholds (from pre-determined maximal treadmill running speeds). While this approach may be time-consuming, recent data suggest that individualized speed thresholds may provide practically significant information regarding training loads [9].

### Neuromuscular Function

Measures of neuromuscular function such as the jump test (countermovement/squat jump), sprint performance, and isokinetic and isoinertial dynamometry are often utilized in the team sport environment [10]. These assessments have become popular due to the simplicity of administration and the minimal amount of additional fatigue induced [10]. Common variables from jump test measurements include mean power, peak velocity, peak force, jump height, flight time, contact time, and rate of force development [5, 10]. Equipment requirements for jump testing may include contact mats, portable or non-portable force platforms, and rotary encoders. As isokinetic and isoinertial dynamometry requires specialized and often expensive equipment and does not replicate sport-specific movements, they are often not utilized in applied settings for strictly monitoring purposes [10].

## Methods of Monitoring Internal Load

### Perception of Effort

The rating of perceived exertion (RPE) is one of the most common means of assessing internal load. The use of RPE is based on the notion that an athlete can monitor their physiological stress during exercise as well as retrospectively provide information regarding their perceived effort post training or competition. Evidence suggests that RPE correlates well with heart rate during steady-state exercise and high-intensity interval cycling training, but not as well during short-duration high-intensity soccer drills [11]. Further, a meta-analysis of the literature reported that while RPE is a valid means of assessing exercise intensity, the validity may not be as high as previously thought [12]. For example, weighted mean validity coefficients for heart rate (HR), blood lactate, and percent of maximal oxygen uptake (*V*O_2max_) were 0.62, 0.57, and 0.64, respectively [12]. RPE is also often combined with other variables such as session duration, HR, and blood lactate to provide additional insights into the internal load experienced by the athlete.

### Session Rating of Perceived Exertion (RPE)

Foster [13] developed the session RPE method of quantifying training load, which involves multiplying the athlete’s RPE (on a 1–10 scale) by the duration of the session (in minutes). This simple method has been shown to be valid and reliable, with individual correlations between session RPE and summated HR zone scores ranging between *r* = 0.75 and *r* = 0.90 [13]. Subsequent research in soccer training has identified individual correlations between RPE and HR zones (range from *r* = 0.54 to *r* = 0.78) and a correlation of *r* = 0.84 has also been reported in endurance athletes [14]. The session RPE method was developed to eliminate the need to utilize HR monitors or other methods of assessing exercise intensity. While the session RPE method may be simple, valid, and reliable, the addition of HR monitoring may aid in understanding some of the variance that it does not explain.

### Heart Rate (HR)

Monitoring HR is one of the most common means of assessing internal load in athletes. The use of HR monitoring during exercise is based on the linear relationship between HR and the rate of oxygen consumption during steady-state exercise [15]; however, percentage of maximum HR is often used to both prescribe and monitor intensity [14]. Due to the daily variation in HR, which may be up to 6.5 % for submaximal HR [16], controlling for factors such as hydration, environment, and medication is important.

### HR to RPE Ratio

Examination of physiological and perceptual indicators of load at a fixed submaximal intensity can provide information on the state of fatigue of the athlete. The combination of HR and perception of effort measures (HR–RPE ratio) may aid in elucidating fatigue [17]. For example, the internal load of a cyclist who has a reduced submaximal HR in combination with an elevated RPE, may be quite different from a cyclist with a normal HR–RPE ratio [1].

### Training Impulse (TRIMP)

The training impulse (TRIMP) is often considered a useful means of assessing training load [1]. A TRIMP is a unit of physical effort that is calculated using training duration and maximal, resting, and average HR during the exercise session [18]. Further derivations of Banister’s initial TRIMP model [19] have been developed. These include Edwards’ TRIMP, which uses accumulated time in five arbitrary HR zones multiplied by a weighting factor [20]. Lucia’s TRIMP model is similar to Edwards’; however, there are three HR zones that are based on individually determined lactate thresholds and onset of blood lactate accumulation [21]. Further, the use of an individualized TRIMP (iTRIMP) has been developed for use in runners [22] and recently tested in soccer players [23]. The use of the iTRIMP reduces issues associated with arbitrary zones and generic weightings and has been shown to relate better than previous TRIMP models to changes in velocity at 2 mmol L^−1^ in professional youth soccer players [23]. However, the authors recognize the technical and scientific expertise and resources required for this type of individualized internal load monitoring.

### Lactate Concentrations

Blood lactate concentration is sensitive to changes in exercise intensity and duration [24]; however, there are a number of potential limitations to the use of regular monitoring of lactate concentrations during training and competition. These include inter- and intra-individual differences in lactate accumulation depending on ambient temperature, hydration status, diet, glycogen content, previous exercise, and amount of muscle mass utilized, as well as sampling procedures (time and site) [14].

### Lactate to RPE Ratio

Similar to the HR–RPE ratio, the lactate to RPE ratio may be useful in determining internal load and identifying fatigue in athletes [25]. Again, changes in these parameters at a fixed submaximal workload may be useful to identify physiological and perceptual changes in internal load.

### HR Recovery (HRR)

HR recovery (HRR) is the rate at which HR declines at the cessation of exercise and has been suggested to be a marker of autonomic function and training status in athletes [26]. The autonomic nervous system consists of the sympathetic and parasympathetic systems, with the rise in HR during exercise being the result of increased sympathetic activity in combination with a reduction in parasympathetic activity. HRR is characterized by opposing autonomic nervous system activity, with an increase in parasympathetic activity and withdrawal of sympathetic nervous activity [27]. HRR can be calculated over varying timeframes, usually between 30 s and 2 min, with the difference between end of exercise HR and HR at 60 s post-exercise being most commonly used [26].

In a recent review on HRR and monitoring changes in training status [26], it is suggested that HRR improves with increased training status, remains unchanged when there is no change in training status, and decreases when training status is reduced. It was then concluded that, with the exception of overreaching (where research is conflicting), HRR could be used to monitor the accumulation of fatigue in athletes [26]. However, the considerations mentioned in Sect. 6.3 regarding standardization of factors that may influence HR are also relevant for HRR.

### HR Variability

The measurement of resting or post-exercise HR variability (HRV) has been suggested to indicate both positive and negative adaptations to training [28]. However, the varying methodological approaches employed, as well as high day-to-day variability in environmental and homeostatic factors, have resulted in inconsistent findings in the scientific literature [28]. As such, HRV has been shown to increase without a change in fitness (*V*O_2max_) [29] as well as decrease alongside increases in fitness [30]. Increases, decreases, and no change in HRV have also been reported in the over-training literature [31]. To overcome some of the inconsistencies in findings, it has been suggested that both weekly and 7-day rolling averages have higher validity than single-day measurements [32]. While various HRV indices can be measured, Plews et al. [28] prefer the use of the natural logarithm of the square root of the mean sum of the squared differences between R–R intervals (Ln rMSSD). This is due to the lower co-efficient of variation compared with other indices, a lack of influence of breathing frequency, and that data can be collected over a short period of time and easily calculated. As is the case with the majority of tools to monitor elite athletes, longitudinal monitoring and an understanding of individual responses in HRV to training, taper and competition is critical.

### Biochemical/Hormonal/Immunological Assessments

A relatively large amount of research has been conducted examining a range of biochemical, hormonal and immunological responses to exercise, primarily in a bid to monitor fatigue and minimize excessive fatigue and illness. It is beyond the scope of this article to review the literature in this area; however, in short, no definitive marker has yet been identified.

Serum creatine kinase activity is often a popular measure due to the simplicity of sample collection and analysis; however, variability of this measure is very high, and a poor temporal relationship with muscle recovery exists [10]. Salivary cortisol and testosterone measures have been shown to have some relationship to performance in the overreached athlete; however, the usefulness of these measures to quantify internal load on a regular basis has not been examined [33]. Other hormonal measures and suggested markers of immune function, such as salivary immunoglobulin A, natural killer cell activity, and neutrophil phagocytic activity have also not been examined on a routine basis, potentially due to both the expense and the time required for analysis [34].

In summary, the use of biochemical, hormonal and/or immunological measures as indicators of internal load is currently not justified based on the limited research in this area. In addition, these measures can be costly, time consuming and impractical in an applied environment [10].

### Questionnaires and Diaries

Questionnaires and diaries can be a relatively simple and inexpensive means of determining the training load and subsequent responses to that training. However, both questionnaires and diaries rely on subjective information, which may need to be corroborated with physiological data [11]. It is possible for athletes to manipulate data and/or over- or underestimate training load. Importantly, the frequency of questionnaire administration and length of questionnaire should be considered to maximize compliance and avoid questionnaire ‘fatigue’. A number of questionnaires are identified in the literature as well as being utilized by high-performance sport programs [5]. These include the Profile of Mood States (POMS) [35], The Recovery-Stress Questionnaire for athletes (REST-Q-Sport) [36], Daily Analysis of Life Demands for Athletes (DALDA) [37], and the Total Recovery Scale (TQR) [38].

While questionnaires can provide simple and often useful subjective information, factors such as frequency of administration, time taken to complete the questions, sensitivity of questionnaire, type of response required (written answers or circling responses), time of day of completion and the amount of time required for appropriate feedback should all be considered.

### Psychomotor Speed

Fatigued athletes often report impaired concentration and cognitive complaints [39]; therefore, investigation into psychomotor speed might provide insight into the cognitive load induced by exercise. Impairments in psychomotor speed following 2 weeks of overload training have been observed in well trained cyclists [40] and in functionally overreached cyclists [41]. Psychomotor speed is most often assessed using computer-based reaction time and rapid visual information processing tasks and therefore can be affordable. While this measure may be applicable for examining overreached athletes, it is yet to receive research attention in the area of determining cognitive load as an indicator of internal load.

### Sleep

Sleep loss or deprivation can have significant effects on performance, motivation, perception of effort and cognition as well as numerous other biological functions [42]. Monitoring sleep quality and quantity can be useful for early detection and intervention before significant performance and health decrements are observed. The use of simple diaries indicating hours of sleep and perceived sleep quality can be useful. Other non-invasive methods such as actigraphy (wrist watch device utilizing accelerometry) can provide more detailed information over shorter periods of 7–14 days. Actigraphy can provide data on bedtime, wake time, sleep-onset latency (time taken to fall asleep), wake during sleep, and sleep efficiency (estimate of sleep quality), as well as provide information on sleep routines. Due to the increasing knowledge regarding the importance of sleep, sleep monitoring and assessment is becoming popular with elite athletes, coaches, and support staff.

## Current Monitoring Practices

Current best practice methods for monitoring fatigue in high-performance sport were recently examined by Taylor [5]. A total of 55 individuals working with high-performance programs across Australia and New Zealand completed an online survey, with 91 % indicating that they implemented some form of training monitoring and a majority (70 %) reporting equal focus on load quantification and monitoring fatigue and recovery within their system. The most important reasons for monitoring were reported to be injury prevention (29 %), monitoring the effectiveness of the training program (27 %), maintaining performance (22 %), and preventing overtraining (22 %) [5]. In terms of the importance of monitoring to the overall performance of the athletes, 38 % of respondents rated it extremely valuable. Self-report questionnaires were the most common means of monitoring fatigue (84 %), with the frequency of monitoring reported as daily (55 %), multiple times per week (24 %), weekly (18 %), or monthly (2 %) [5]. A performance test was used by 61 % of respondents and included tests such as maximal jump and/or strength tests, over-ground sprints, submaximal cycling or running tests and sport-specific tests. These tests were completed either weekly (33 %), monthly (30 %) or more frequently than weekly (daily or multiple times per week; 36 %). Measuring performance during competition was also reported by 43 % of respondents, with GPS monitoring being used by team sports, cyclists and rowers [5]. Finally, hormonal profiling (*n* = 4), musculoskeletal screening (*n* = 1) and resting HR upon waking (*n* = 1) were other monitoring measures utilized.

From this assessment of monitoring, it appears that monitoring is incorporated by many staff in high-performance programs and that self-report measures are most commonly used, followed by practical sport-specific performance assessments. Support staff and coaches are incorporating these techniques regularly, with the goal of minimizing fatigue and injury as well as examining the effectiveness of the training program.

### Team Sport versus Individual Sport Athletes

The nature of load monitoring required or indeed possible may vary greatly between team sport and individual sport athletes. Monitoring in team sports is often perceived to be more challenging due to the diverse range of training activities (e.g. general conditioning, resistance training, interval training and skill-based conditioning) commonly employed. Further, the assessment of skilled performance and ‘cognitive load’ or fatigue that influences decision making is important for team sport performance and poses many challenges for accurate assessment.

When monitoring team sport athletes, some of the most useful measures involve physiological changes, assessment of movement patterns and indicators of skills [1], with these measures being as sport-specific as possible. Movement patterns can be assessed by time-motion analysis or GPS tracking [1]. Other difficulties when assessing team sport competition performance include the influence of team tactics (including those of the opposing team), environmental conditions, team cohesion, home or away competition and travel.

In individual sports such as cycling, swimming and triathlon, the fatigue is often the result of high training loads; the management of these loads through monitoring can be particularly important [1]. Load monitoring is often based on training volume, duration and intensity alongside indicators of perceptual fatigue such as RPE.

## The Importance of Individualized Monitoring

As highlighted in the previous section, there are a number of differences between the requirements for monitoring of team and individual sport athletes. Further, there is also a need to ensure appropriate monitoring of individuals within a team environment. Individual athletes may respond differently to a given training stimulus, and the training load required for adaptation may differ significantly from one athlete to another. Monitoring the individual athlete allows the identification of those athletes who are not responding to the training program and where there may be a disassociation between external and internal loads.

An individualized approach is also important to ensure that the internal load experienced by the athlete corresponds with that intended by the coach. Wallace et al. [6] assessed the ecological validity of the session-RPE method to quantify internal training load when compared with HR and distance swum. One of the findings of the study when examining the athlete’s and coach’s perception of internal load using the session-RPE method was a tendency for athletes to report higher training intensities than coaches during sessions designed to be easy. Further, lower training intensities were reported during sessions designed to be difficult [6]. Thus, individual monitoring of load can be useful to ensure the load applied is matched to that which the coach prescribes.

## Assessing Meaningful Change

The determination of whether changes observed when monitoring training are clinically or practically relevant is of particular importance. The use of magnitude-based inferences with reference to sport-specific thresholds is becoming popular in the scientific literature and with applied practitioners in the field [1]. Knowledge of the smallest worthwhile change (SWC) and typical error of measurement allows confidence when making decisions about the meaningfulness of any observed changes [5] and whether these changes should be acted upon.

Twist and Highton [10] suggest that, due to the differences in SWC and the variable reliability of different measures, arbitrary cut-off points, such as a change greater than 5%, should not be used. Identifying the reliability of each measure (co-efficient of variation), the SWC and expressing change in effect sizes can aid in detecting a meaningful change. This approach can add scientific legitimacy to the monitoring approach as well as allow the expression of data in a meaningful manner to athletes and coaches.

## Utilizing a Systems-Based Approach

With the increasing amounts of data available from monitoring devices such as GPS, digital video and SRM devices, in combination with internal load measurements such as HR, questionnaires and perceptions of fatigue comes the requirement to incorporate this information into a database and data-management system that results in efficient access to meaningful information. According to Pyne and Martin [1], “a systems-based approach that integrates well-chosen diagnostic tests, with smart sensor technology and a real-time database and data management system, is the future for fatigue management in elite sport.” There are now a number of commercially available athlete monitoring systems such as Training Peaks™, Kinetic Athlete and Smartabase that allow for integration of data, and simple reporting tools that are becoming increasingly popular in high-performance sport.

### Specific Example

**Fig. 1:**
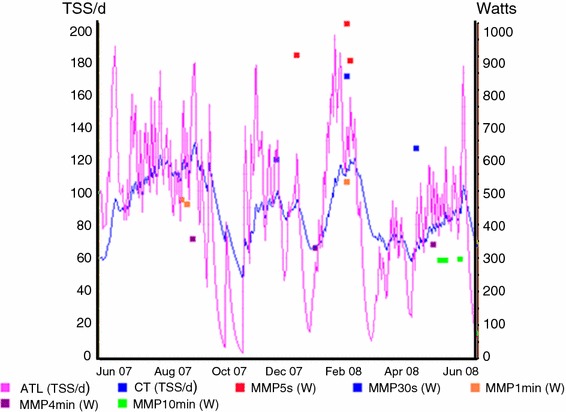
The Training Stress Score™ of an elite female cyclist over a 12-month period. The *blue line* depicts a long-term rolling average (20 days) and indicates fitness CTL. The *pink line* is a 5-day rolling average and indicates fatigue ATL. Maximal mean power for specified durations are also shown, with the highest three MMPs for 5, 30 s, 1, 4, and 10 min averaged over 24 months highlighted. *ATL* acute training load, *CTL* chronic training load, *MMP* mean maximal power, *TSS* Training Stress Score™. Reproduced with permission from Nikki Butterfield

Figure 1 depicts the TSS™ of an elite female cyclist over a 12-month period. The Training Peaks TSS is a training load index that takes into account the duration and intensity of a workout based on power output. It is conceptually modeled after the HR-based TRIMP. By definition, 1 h spent at functional threshold power (FTP) is equal to 100 points. The TSS™ can be used to understand patterns by calculating short- and long-term rolling averages to reflect fatigue and fitness.

In 1980, Eric Banister proposed a means of modeling performance based on assessments of fitness and fatigue [19]. Fitness is considered a positive influence on performance and is both slow to develop and slow to dissipate [1]. However, fatigue can occur quickly and dissipate more rapidly. Figure 1 is a graphical representation of daily power of an elite female cyclist over a 12-month period. Power data were collected using an SRM™ device and analysed utilizing Training Peaks™ software. These data can be examined over time to highlight when personal best performances occur and to gain an understanding of when athletes may be likely to produce exceptional performances [1].

## Key Features of a Sustainable Monitoring System

An effective and sustainable monitoring system is critical to ensure data are effectively captured and reported. Table 2 below identifies several key features of such a system.

**Table 2:** Key features of a sustainable monitoring system

Ease of use/intuitive design
Efficient result reporting
Can be used with or without internet connection, i.e. able to be utilized effectively remotely
Data should be able to be translated into simple outcomes, such as effect sizes
The system should be flexible and adaptable for different sports and athletes
Identification of a meaningful change should be simple and efficient
Should include an assessment of cognitive function
Should be able to provide both individual responses and group responses

## Conclusions

Despite both the increasing amounts of research and the popularity of load monitoring in high-performance programs, a single definitive tool that is accurate and reliable is not evident. Indeed, the nature of the monitoring is likely to be very different depending on the sport and more than one monitoring tool is often utilized. This is likely the consequence of individual physiological adaptation and responses to exercise as well as the specificity required to be relevant to differing sports. However, recent evidence suggests that many athletes, coaches and support staff are taking an increasingly scientific approach to load monitoring.

Utilizing scientific principles for load monitoring can be an important means of reducing the risk of non-functional overreaching, illness, and injury. With many athletes exposed to high training loads and high training and competition stress, it is necessary to manage risks associated with the possible negative outcomes and to maintain optimal physiological and psychological health and well-being of the athlete. While a range of potential measures of external and internal load have been described, numerous factors are involved in determining the reasons for and against load monitoring, the specific type of monitoring necessary for the sport and the individual and ensuring change is evaluated in an appropriate manner. If accurate and easy-to-interpret feedback is provided to the athlete and coach, load monitoring can result in enhanced knowledge of training responses, aid in the design of training programs, provide a further avenue for communication between support staff and athletes and coaches and ultimately enhance an athlete’s performance.
